# Supplementary material for: Efficacy Comparison between Kegel Exercises and Extracorporeal Magnetic Innervation in Treatment of Female Stress Urinary Incontinence: A Randomized Clinical Trial
Source: Medicina (Kaunas). 2022 Dec 17;58(12):1863. doi: 10.3390/medicina58121863 (PMC9784675; doi:10.3390/medicina58121863)
Supplement: Supplementary file 1 [file medicina-58-01863-s001.zip › medicina-2020337-supplementary.pdf]

Supplemental Files

Table S1. Analysis of repeated measurements for the variable intravaginal pressure for the EMI group.

		M_1-M_2
No. 1	No. 2	-7.176*
	No. 3	-6.411*
No. 2	No. 1	7.176*
	No. 3	.765
No. 3	No. 1	6.411*
	No. 2	-.765

No. 1 – first measurement (starting point), No. 2 – second measurement (8 weeks), No. 3 – third measurement (3 months), M_1-M_2 – difference of mean measurement values.
*$p<0.005$.

Table S2. Analysis of repeated measurements for the variable intravaginal pressure for the Kegel group.

		M_1-M_2
No. 1	No. 2	-3.350
	No. 3	-2.394
No. 2	No. 1	3.350
	No. 3	.956
No. 3	No. 1	2.394
	No. 2	-.956

No. 1 – first measurement (starting point), No. 2 – second measurement (8 weeks), No. 3 – third measurement (3 months), M_1-M_2 – difference of mean measurement values.

Table S3. Analysis of repeated measurements for the variable ICIQ-UI SF for the EMI group.

		M_1-M_2
No. 1	No. 2	5.065*
	No. 3	5.543*
No. 2	No. 1	-5.065*
	No. 3	.478
No. 3	No. 1	-5.543*
	No. 2	-.478

No. 1 – first measurement (starting point), No. 2 – second measurement (8 weeks), No. 3 – third measurement (3 months), M_1-M_2 – difference of mean measurement values.
*$p<0.005$

Table S4. Analysis of repeated measurements for the variable ICIQ-UI SF for the Kegel group.

		M_1-M_2
No. 1	No. 2	2.083*
	No. 3	1.813
No. 2	No. 1	-2.083*
	No. 3	-.271
No. 3	No. 1	-1.813
	No. 2	.271

No. 1 – first measurement (starting point), No. 2 – second measurement (8 weeks), No. 3 – third measurement (3 months), M_1-M_2 – difference of mean measurement values.
*p<0.005.

Table S5. Analysis of repeated measurements for the variable ICIQ-LUTSqol for the EMI group.

		M_1-M_2
No. 1	No. 2	17.370*
	No. 3	21.196*
No. 2	No. 1	-17.370*
	No. 3	3.826
No. 3	No. 1	-21.196*
	No. 2	-3.826

No. 1 – first measurement (starting point), No. 2 – second measurement (8 weeks), No. 3 – third measurement (3 months), M_1-M_2 – difference of mean measurement values.
*p<0.005.

Table S6. Analysis of repeated measurements for the variable ICIQ-LUTSqol for the Kegel group.

		M_1-M_2
No. 1	No. 2	8.021*
	No. 3	7.771*
No. 2	No. 1	-8.021*
	No. 3	-.250
No. 3	No. 1	-7.771*
	No. 2	.250

1 – first measurement (starting point), 2 – second measurement (8 weeks), 3 – third measurement (3 months), M_1-M_2 – difference of mean measurement values.
*p<0.005.

Table S7. Analysis of repeated measurements for the variable bladder diary for the EMI group.

		M_1-M_2
No. 1	No. 2	5.696*
	No. 3	5.630*
No. 2	No. 1	-5.696*
	No. 3	-.065
No. 3	No. 1	-5.630*
	No. 2	.065

No. 1 – first measurement (starting point), No. 2 – second measurement (8 weeks), No. 3 – third measurement (3 months), M_1-M_2 – difference of mean measurement values. *$p<0.005$.

Table S8. Analysis of repeated measurements for the variable bladder diary for the Kegel group.

		M_1-M_2
No. 1	No. 2	1.250
	No. 3	1.250
No. 2	No. 1	-1.250
	No. 3	.000
No. 3	No. 1	-1.250
	No. 2	.000

No. 1 – first measurement (starting point), No. 2 – second measurement (8 weeks), No. 3 – third measurement (3 months), M_1-M_2 – difference of mean measurement values. *$p<0.005$.

Table S9. Overall satisfaction with treatment between groups.

	EMI Group	Kegel Group	p
PGI-I	2.30±1.01	3.72±1.31	<0.001

PGI-I – Patient Global Impression of Improvement.
